# Patterns of perception toward influenza pandemic among the front-line responsible health personnel in southern Thailand: a Q methodology approach

**DOI:** 10.1186/1471-2458-9-161

**Published:** 2009-05-28

**Authors:** Tapanan Prateepko, Virasakdi Chongsuvivatwong

**Affiliations:** 1 Epidemiology Unit, Faculty of Medicine, Prince of Songkla University, Hatyai, Thailand

## Abstract

**Background:**

Thailand has joined the World Health Organization effort to prepare against a threat of an influenza pandemic. Regular monitoring on preparedness of health facilities and assessment on perception of the front-line responsible health personnel has never been done. This study aimed to document the patterns of perception of health personnel toward the threat of an influenza pandemic.

**Methods:**

Q methodology was applied to a set of 385 health personnel in charge of influenza pandemic preparedness in the three southernmost provinces of Thailand. Subjects were asked to rank 33 statements about various issues of influenza pandemic according to a pre-designed score sheet having a quasi-normal distribution on a continuous 9-point bipolar scale ranging from -4 for strongly disagree to +4 for strongly agree. The Q factor analysis method was employed to identify patterns based on the similarity and dissimilarity among health personnel.

**Results:**

There were three main patterns of perception toward influenza pandemic with moderate correlation coefficients between patterns ranging from 0.37 to 0.55. Pattern I, health personnel, which we labeled pessimistic, perceived themselves as having a low self-efficacy. Pattern II, which we labeled optimistic, perceived the threat to be low severity and low vulnerability. Pattern III, which we labeled mixed, perceived low self-efficacy but low vulnerability. Across the three patterns, almost all the subjects had a high expectancy that execution of recommended measures can mitigate impacts of the threat of an influenza pandemic, particularly on multi-measures with high factor scores of 4 in all patterns. The most conflicting area was vulnerability on the possible impacts of an influenza pandemic, having factor scores of high (3), low (-4), and neutral (0) for patterns I, II, and III, respectively.

**Conclusion:**

Strong consistent perceptions of response efficacy against an influenza pandemic may suggest a low priority to convince health personnel on the efficacy of the recommended measures. Lack of self-efficacy in certain sub-groups indicates the need for program managers to improve self-confidence of health personnel to participate in an emergency response.

## Background

An influenza pandemic is a significant natural health threat that has periodically occurred over the past 300 years [1]. Its severe impacts to global human health, healthcare service, society, and economy were evidently documented during the previous pandemics [2,3]. For a coming one, influenza experts have agreed that this threat is inevitable and possibly imminent [4]. If the next pandemic occurs, it is expected that 20% of the global population will become ill, nearly 30 million will be hospitalized and a quarter of these would die within a few months of its attack [5]. To mitigate the impacts of this threat, the World Health Organization (WHO) has recommended that all countries should consider this threat as very important and urged them to make preparations a high national priority.

Thailand occasionally has had serious outbreaks of avian influenza A (H5N1) since early 2004, in both poultry and humans. In response to these outbreaks and a possible future influenza pandemic, the national committee on avian influenza control and influenza pandemic preparedness has issued a national strategic plan for influenza pandemic preparedness.

Beyond preparedness, the perception of each individual is also a fundamental factor that contributes to the spread, prevention, and control of infectious diseases. For example, during the Severe Acute Respiratory Syndrome (SARS) epidemics, the perceptions toward this disease had an effect on the preventive health behaviors (e.g., hand hygiene, mask wearing) and that consequently contributed to containing the outbreaks [6-8]. For a current threat of an influenza pandemic, sporadic perception surveys among health workers have been done in developed countries [9-12]. Yet this issue has not been explored in developing countries, particularly in the southeast Asian region where it is more likely to be a source of the next pandemic [13].

Southern Thailand experienced a probable SARS case in 2003, but there has been no reported case of avian influenza A (H5N1) in both poultry and human. However, the region faces a serious problem of ethnic violence. This unrest has led to the loss of over 2,600 lives and more than 7,000 injuries in the past 5 years. It is possible that the local health systems may have deteriorated due to the unrest leading to loosening of preparedness against the threat of an influenza pandemic. We have therefore conducted a study to investigate the preparedness. The current report is confined to perceptions related to the threat of an influenza pandemic with the objective to document the patterns of perception of health personnel toward this threat in southern Thailand. As health personnel are key persons for influenza pandemic preparedness and control, it is hoped that understanding their patterns of perception will allow control programs to properly improve the training. It may also be useful for other developing countries where an influenza pandemic is a serious threat, but the personnel are not fully prepared.

## Methods

### Study design

Q methodology, which basically originated from the theory of factor analysis [14] was applied. While conventional factor analysis is used in scale development and tries to group items or variables, Q method tries to group subjects. Therefore, people of the same group or having the same factor will have a similar pattern of chosen statements. The implication would be that it would be easy to put people of the same factor into the same intervention program. This method was taken into our study because this is a scientific and systematic study of human subjectivity, involving perceptions, attitudes, and opinions [15,16]. Furthermore, it is also unique since it mixes the strong points of both qualitative and quantitative research techniques, compared to traditional surveys [17,18].

In doing Q, the flow of communication surrounding the study topic (concourse) is firstly formed to get a wide range of ideas toward that topic. This is generally collected from various sources (e.g., scientific papers, books, news, interviews, focus group discussions, etc.). It is commonly presented in the form of statements. Afterward, a Q sample (a representative set of statements) is selected from the concourse and developed to be more meaningful, which represents various issues of the study topic and eventually is compiled into the instrument. The study subjects are then asked to rank the representative statements and place them into a score sheet, which is designed in a continuous scale ranging from strongly disagree to strongly agree, following a standardized instruction based on the judgment of each subject. This is known as the Q sorting procedure. The sheets that are completely ranked by each subject (Q-sort) are finally correlated and analyzed by Q (subject-wise) factor analysis, and the factors are then interpreted.

**Figure 1:**
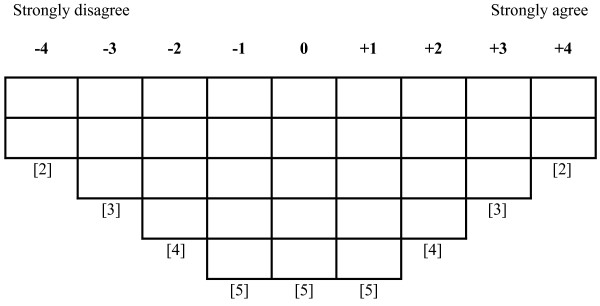
**Participant score sheet**.

In our study, 100 statements on various issues of an influenza pandemic were initially gathered from scientific articles, newsletters, and books to form a concourse. The protection motivation theory (PMT) was used as a basis for grouping and developing the statements into four domains: perceived severity, perceived vulnerability, perceived response efficacy, and perceived self-efficacy, by refining, clarifying, and combining the raw statements to be more meaningful and more understandable. To catch various aspects of an influenza pandemic and keeping the total number of the statements suitably manageable by our subjects, we included eight refined statements in each of such four domains with one additional item added to make the total number of the statements equal 33 (Q-sample). These statements were then placed into the score sheet (Figure 1), and forced to follow a quasi-normal distribution, that is, 2-3-4-5-5-5-4-3-2. The reliability of this instrument was tested with Cronbach's alpha. Each statement was randomly assigned a number from 1 to 33 for the subjects to arrange and place into the score sheet. To get more understandability, the statements were pilot-tested with 25 health personnel and were then revised as appropriate before the study.

### Study setting

The study was conducted in the three southernmost provinces of Thailand: Yala, Pattani, and Narathiwat, during April to October 2007. Apart from the problems of ethnic violence, the area is in a remote part of the country where the logistic problems will be easily visible. The area is also close to Malaysia, so cross-border diseases have a high chance of spread due to the movement of populations.

### Study subjects and procedures

The research protocol was approved by the Ethics Committee of the Faculty of Medicine, Prince of Songkla University, prior to conducting the study. A list of all health facilities in the study area was obtained from the local health offices. Health personnel designated by each facility to be responsible for influenza pandemic preparedness were identified. These included a numbers of doctors, nurses, pharmacists, laboratory personnel, public health specialists, public health administrators, and junior health workers. All were invited to participate in the study. The selected personnel were sent a set of documents, which included a cover letter, an overview describing the study importance and objective, a set of 33 statements (Q sample), a standardized step-by-step set of instructions for responding to the study, and a score sheet. Following the initial mailing, two phases of follow up were performed: a sequence of telephone calls at one month, with non-responders contacted by the first author after three months.

Each consenting subject was asked to rank the 33 statements about different issues concerned with an influenza pandemic into the levels of agreement and disagreement based on their own judgments. Each participant was requested to place two statements in the columns of strongly disagree (-4) and strongly agree (+4), three in disagree (-3) and agree (+3), four in -2 and +2, five in -1 and +1, and five statements in the neutral response column (0). However, if they thought that our distribution did not represent their real perceptions, they were encouraged to sort such statements accordingly. Each Q-sort was considered as complete if all 33 statements were placed into the score sheet without repetition of the statements.

### Data entry and analysis

**Figure 2:**
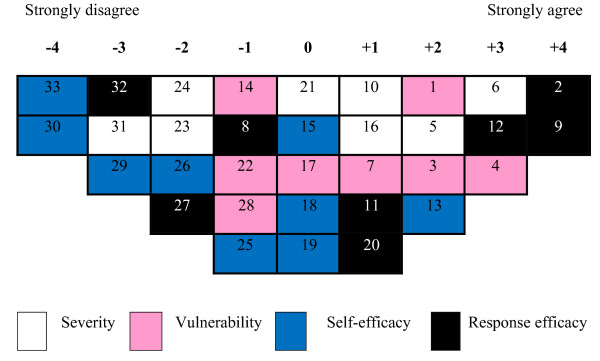
**Pattern I. pessimistic with perceived low self-efficacy**.

**Figure 3:**
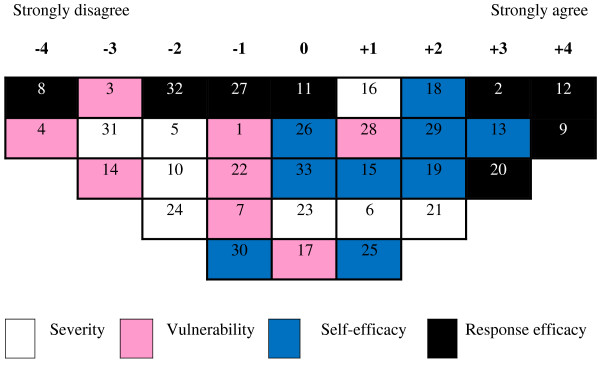
**Pattern II. optimistic with perceived low severity and low vulnerability**.

**Figure 4:**
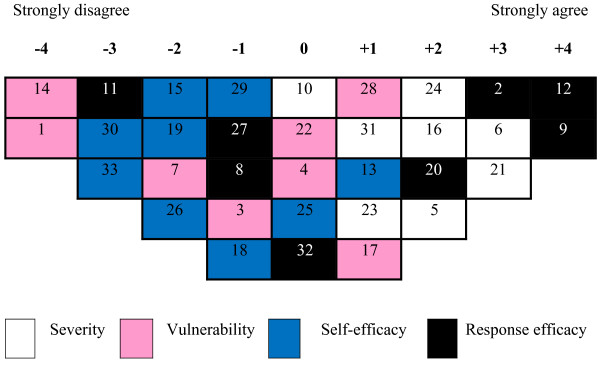
**Pattern III. mixed with perceived low self-efficacy but low vulnerability**.

The data from each completed score sheet were entered and analyzed in PQMethod 2.11 (free software). Between-subjects correlation matrix was computed and a Q (subject-wise) factor analysis by principle components analysis (PCA) method was performed using a varimax rotation technique. Factors that could explain more than 5% of the variance were adopted and retained into the final solution. A participant who had absolute factor loadings of larger than ± 0.45, which suggests high significance (p < 0.01) with the group, was included into that particular factor. In each factor, the ascending sorted normalized scores (Z-scores) of assigned number of each statement were returned into the score sheet from right to left order (Figures 2, 3, and 4). Each final score sheet thus displays the pattern of the defined factor. Comparisons among patterns were based on the factor scores and the mean values of the domain of the statements. For visualization of the patterns, the domains of each statement were linked to different colors or grey shadings in the final Q-sort models that are shown in Figures 2, 3, and 4. Since the cells in the extreme score regions reflect strong perceptions in the domains, they are the primary target for comparing similarity and dissimilarity of each group of health personnel's perceptions on the threat of an influenza pandemic.

## Results

After consultation with an expert in instrument development, 33 statements listed in Table 1 were used in the study. Although some statements may resemble others, they measure different aspects or domains on an influenza pandemic. For example, statements 3, 14, and 22 are all concerned with vulnerability (V), but measure or emphasize different aspects. Statement 3 emphasizes avian influenza, statement 14 natural and inevitable threat, and 22 on proximity to the threat. Statements 11 and 19 measure different domains of the PMT: statement 11 represents response efficacy (RE); whereas, statement 19 represents self-efficacy (SE). The subjects should perceive these statements as different questions. The overall Cronbach's alpha of this instrument was 0.70.

**Table 1:** List of statements and composite factor scores by pattern

		Pattern		
		
No.	Statement	I	II	III
1	I perceive that Thailand can be the source of an influenza pandemic since there are many cases of avian influenza A(H5N1) in both humans and poultry in many parts of the country (V)	2	-1	-4
2	I perceive that influenza pandemic preparedness has short and long terms benefits in reducing the impacts of an influenza pandemic, as well as the other emerging infectious diseases (RE)	4	3	3
3	I perceive that there is a high possibility of an occurrence of the next influenza pandemic since there are many cases of avian influenza A(H5N1) in humans and poultry in many parts of the world (V)	2	-3	-1
4	I perceive that Thailand will be affected greatly by an influenza pandemic if and when it occurs (V)	3	-4	0
5	I perceive that an influenza pandemic can cause significant pressure on health care services for several months (S)	2	-2	2
6	I perceive that an influenza pandemic will cause enormous economic loss (S)	3	1	3
7	I perceive that the occurrence of an influenza pandemic cannot be predicted (V)	1	-1	-2
8	I perceive that public health measures (e.g., surveillance, infection control, isolation and quarantine, etc.) have no efficiency in reducing the impacts of an influenza pandemic (RE)	-1	-4	-1
9	I perceive that multi-measures (pharmaceutical and non-pharmaceutical) must be performed during an influenza pandemic event to reduce the impacts (RE)	4	4	4
10	I perceive that an influenza pandemic can causes excess of illnesses, hospitalizations and deaths (S)	1	-2	0
11	I perceive that antiviral drugs are efficient at reducing the impacts of an influenza pandemic (RE)	1	0	-3
12	I perceive that efficient influenza pandemic preparedness is the responsibility of every level from national to community both governmental and private sectors, in order to reduce its impacts. (RE)	3	4	4
13	I have confidence that public health measures (e.g., surveillance, infection control, isolation and quarantine, etc.) are efficient in reducing the impacts of an influenza pandemic (SE)	2	3	1
14	I perceive that an influenza pandemic is an inevitable natural health threat (V)	-1	-3	-4
15	I have confidence that vaccination measures can reduce the impacts of an influenza pandemic (SE)	0	1	-2
16	I perceive that when an influenza pandemic occurs, it will affect all countries around the globe. (S)	1	1	2
17	I perceive that everyone has a high chance to be infected with the virus when a pandemic occurs (V)	0	0	1
18	I have confidence that local health personnel have the capacity to control an influenza pandemic and reduce its impact (SE)	0	2	-1
19	I have confidence that antiviral drug measure can reduce the impacts of an influenza pandemic (SE)	0	2	-2
20	I perceive that performing multi-measures (pharmaceutical and non-pharmaceutical) during an influenza pandemic event can reduce the impacts (RE)	1	3	2
21	I perceive that an influenza pandemic can causes a very high health care cost (S)	0	2	3
22	I perceive that our world is now close to the next influenza pandemic (V)	-1	-1	0
23	I perceive that if an influenza pandemic occurs, every community has to rely on its own resources (help cannot be easily shifted from one community to another), it is not like other natural health threats (S)	-2	0	1
24	I perceive that an influenza pandemic can cause great psychosocial disruption (S)	-2	-2	2
25	I have confidence that I can get influenza vaccines and antiviral drugs when an influenza pandemic occurs (SE)	-1	1	0
26	I have confidence that Thailand has the chance to use vaccines and antiviral drugs when an influenza pandemic occurs (SE)	-2	0	-2
27	I perceive that influenza vaccines have the highest efficiency in reducing the impacts of an influenza pandemic (RE)	-2	-1	-1
28	I perceive that increasing globalization (transportation, communication, urbanization) can cause the rapid spread of an influenza pandemic (V)	-1	1	1
29	I have confidence that Thailand can control an influenza pandemic if and when it occurs (SE)	-3	2	-1
30	I have confidence that influenza vaccines and antiviral drugs will be enough provided for everyone if and when an influenza pandemic occurs (SE)	-4	-1	-3
31	I perceive that an influenza pandemic will cause a great productivity loss (S)	-3	-3	1
32	I perceive that influenza vaccines are cost-effective in reducing the impacts of an influenza pandemic (RE)	-3	-2	0
33	I have confidence that local health personnel can control an influenza pandemic if and when it occurs (SE)	-4	0	-3

S = severity, V = vulnerability, RE = response efficacy, SE = self-efficacy

Of a total 385 health personnel, 271 (70%) persons completed the score sheet. There were no statistically significant differences between responders and non-responders in terms of gender, age, religion, educational level, total period of working, job classification, experience of getting training on influenza pandemic preparedness and perceived levels of knowledge about an influenza pandemic, public health measures against an influenza pandemic and impacts of an influenza pandemic. However, the non-responders had a lower educational level than those of the responders (35% vs. 18%, respectively). The basic characteristics of the 271 respondents are presented in Table 2.

**Table 2:** Basic characteristics of the respondents

Variable	Number (n = 271)	%
**Sex**		
Male	148	54.6
Female	123	45.4
**Age **Mean(SD)	37.4 (8.3)	
**Job classification**		
Public health specialist	92	33.9
Public health administrator	51	18.8
Junior health worker	50	18.4
Nurse	39	14.4
Doctor	14	5.2
Pharmacist	11	4.1
Laboratory personnel	9	3.3
Other	5	1.8
**Educational level**		
Lower than bachelor degree (certificate)	48	17.7
Bachelor degree	202	74.5
Higher than bachelor degree	21	7.8
**Place of work**		
Provincial public health office	8	3.0
Hospital	76	28.0
District public health office	58	21.4
Health center	129	47.6

Q factor analysis gave three factors that met our criteria with the percentages of explained variance being 30.1%, 8.7%, and 5.5%, respectively. After varimax rotation, 90 subjects were classified into factor I (in other words, the first pattern composites of 90 health personnel), 40 into factor II, and 62 into factor III. The other 79 subjects were not classified into any factor because all their loading values were less than 0.45 or had high loading on more than one factor. The composite reliability of each factor was 0.99, with the corresponding standard errors of factor scores being 0.05, 0.08, and 0.06. The correlation coefficients between the three factors were 0.37 (factor I vs. II), 0.54 (factor I vs. III), and 0.55 (factor II vs. III), indicating a moderate similarity among the patterns.

The three patterns had scores for each specific statement distributed into the Q-sort model or composite factor array and are displayed in Figures 2, 3, and 4. The same information is displayed in Table 1. Factor scores of statement 1 were 2, -1, and -4 as shown in the first row of Table 1. In the Q-sort model, statement 1 is in column +2 of Figure 2, and column -1 of Figure 3, and column -4 of Figure 4.

From Table 1, statement number 9 has a common factor score of 4 for all three patterns. This indicates that all three patterns of health personnel strongly perceived that multi-measures must be performed during an influenza pandemic. Statement number 12 was also in columns +4 of Figures 3 and 4, and +3 of Figure 2, which is related to response efficacy on multilevels of responsibility for preparedness against the threat. In contrast, statement 4 was the most dissenting issue with factor scores of 3, -4, and 0. Health personnel classified as pattern I quite strongly perceived that Thailand will have possibly high impacts from an influenza pandemic if and when one occurs, but those classified in pattern II strongly disagreed, and those in the remaining group were neutral.

The right extremes of all three Q-sort models are consistently filled with three black cells (statements 2, 9, and 12) out of 5 cells of that region. This indicates that all three groups of health personnel have strong perception on response efficacy of the control measures rather than on the other domains. The left extremes of those three patterns, on the other hand, contain different mixtures for different patterns. Health personnel classified into pattern I were pessimistic. They had negative perceptions of self-efficacy as there are three blue or dark grey cells (statements 33, 30, and 29) in the columns of -4 and -3. Health personnel classified into pattern III were less extreme about this, but still have two blue (dark grey) cells (statements 30 and 33) in the -3 column. None of the blue cells (dark gray in black-and-white printing) are present in the left extreme regions of the pattern II indicating optimism of the group of personnel.

Means of factor scores for each component of the PMT are displayed in Table 3. All groups had positive perceived response efficacy of the measures. Patterns I and III, however, perceived low self-efficacy, in contrast to high perceived self-efficacy of pattern II.

**Table 3:** Mean factor scores of each component of the PMT by pattern

	Pattern		
	
Component	Pessimistic (n = 90)	Optimistic (n = 40)	Mixed (n = 62)
Perceived severity	0	-0.62	1.75
Perceived vulnerability	0.62	-1.50	-1.12
Perceived response efficacy	0.87	0.87	1
Perceived self-efficacy	-1.33	1.11	-1.44

Optimistic personality of pattern II was also expressed as perception of low severity and low vulnerability where the pattern I has isolated neutral perception of severity with a moderate level of perceived vulnerability. Finally, more mixed appraisal is found in pattern III, the group who perceived a low level of vulnerability but a very high level of severity.

## Discussion

We identified three main patterns of health personnel in southern Thailand based on the perception toward a threat of an influenza pandemic. Pattern I was pessimistic (strongly perceived response efficacy, but perceived low self-efficacy). Pattern II was optimistic (strongly perceived response efficacy, but perceived low severity and low vulnerability). Pattern III was mixed (strongly perceived response efficacy, but perceived low vulnerability and low self-efficacy). A high perception on response efficacy was predominantly found in all health personnel groups. Perceptions on vulnerability were more varied.

The majority of our health personnel perceived low self-efficacy toward an influenza pandemic. Self-efficacy is one important component of coping appraisal of the PMT [19]. It has powerful influence on human's feeling, thinking, motivation, and behavior [20-22]. Previous meta-analyses provided evidence for self-efficacy having the largest effect size and was the strongest predictions of protection motivation [23,24]. People with low self-efficacy usually believe that tasks are harder than they can handle. This can lead to limit task planning, increase stress, reduce the low level of attempt, and having a tendency to avoid duties and activities [20-22]. Balicer *et al. *reported that nearly a half of local health workers may be unwilling to report to duty during a pandemic event [9]. However, that study did not identify different patterns of health workers as our study has done. Another conventional survey conducted among a general population (rather than health workers) in developed countries of Europe and Asia on avian influenza risk perception showed a similar result. The level of self-efficacy among the respondents was also low and the authors concluded that a low level of self efficacy may obstruct any interventions [25].

The most dissenting issue among our health personnel toward this threat was on vulnerability of possible impacts in the country (statement number 4). Naturally, the occurrence and severity of an influenza pandemic cannot be predicted [26]. Fifteen per cent of our health personnel perceived the threat to have low severity and low vulnerability. In other words, this group of health personnel was optimistic that such a threat would not severely affect people's lives. A small survey by Curtis *et al. *on the perceptions toward such a threat among physicians showed that more than half did not consider that the risk of an imminent influenza pandemic was more than a possibility [10]. Both perceived severity and perceived vulnerability are components of threat appraisal of the PMT [19]. Perception of low level of severity and vulnerability or low levels of appraised threat of an influenza pandemic may inhibit motivation of health personnel to engage in protective behavior [27,28]. However, the effect sizes of such two components in previous meta-analyses were small to medium and barely predicted of protection motivation and behavior compared to the components of coping appraisal (response efficacy and self-efficacy) [23,24].

Perception of response efficacy was stronger than other domains. This may be influenced by past experiences of the country, which after employing on multi-sectors and multi-measures could successfully suppress avian influenza A(H5N1) [29].

This study used a wide range of front-line health personnel responsible for influenza pandemic preparedness. Thus, it may reflect the problems specific to this area with acceptable accuracy. The study was confined to the three southernmost provinces of Thailand where avian influenza A (H5N1) has never occurred. Our study subjects might be different from those in other regions of the country where the infected cases of that avian influenza in both humans and poultry have been reported, and intensive avian influenza controls have been fully activated. The study subjects were also predominated by personnel from health centers and community hospitals in rural areas. The threat of a pandemic may be less compared to in urban areas. The study was based on Q methodology which had never been employed among local health workers; thus, the data have to be interpreted with caution. Approximately 30% of the respondents were not able to be classified into any of the three groups determined by our factor analysis. The patterns are therefore far from ideal. The statements about influenza pandemic that were used in our study should be improved to be more specific for health workers in future work.

## Conclusion

Despite the above limitations, this study highlights important findings. Strong consistent perceptions of implementing recommended measures against an influenza pandemic can remove or mitigate impacts of this threat, and may suggest a low priority to convince health personnel on the efficacy of the measures. Perception of low self-efficacy in certain subgroups who gave low scores on the statements related to self-efficacy on an influenza pandemic indicates the need to improve self-confidence of health personnel to participate in an emergency response by the control program.

## Competing interests

The authors declare that they have no competing interests.

## Authors' contributions

TP designed this study, was the principal investigator of the project, performed data analysis, and drafted the manuscript. VC provided supervision, suggestion, and development on manuscript writing. All authors have contributed to revision of the draft version and have read and accepted the final version of this manuscript.

## Pre-publication history

The pre-publication history for this paper can be accessed here:


